# The relation between intensive parenting attitude and preschoolers’ social–emotional competence: the chain mediating role of family functioning and psychological resilience

**DOI:** 10.3389/fpsyg.2026.1643840

**Published:** 2026-04-10

**Authors:** Haiping Wang, Yi Zhang

**Affiliations:** 1 College of Education, Open University of China, Beijing, China; 2 Department of Sociology, Peking University, Beijing, China

**Keywords:** family functioning, intensive parenting attitude, preschoolers, psychological resilience, social–emotional competence

## Abstract

**Introduction:**

Social-emotional competence is crucial for preschoolers’ development. While previous research has emphasized the importance of parenting styles, little attention has been paid to how multidimensional intensive parenting attitudes and interaction between individuals and families jointly promote the development of social-emotional competence. Grounded in social–ecological systems Theory, the present study investigated the association between intensive parenting attitude and preschoolers’ social–emotional competence, with particular attention to the chain-mediated role of family functioning and psychological resilience.

**Methods:**

A total of 2,174 preschoolers aged 3 to 6 years from four provinces in China were assessed through parent-report questionnaires using standardized scales: the Intensive Parenting Attitudes Questionnaire, the Chinese Inventory of Children’s Social-emotional Competence, the Family Functioning Assessment Scale, and the Devereux Early Childhood Assessment for Preschoolers.

**Results:**

Regression-based chain mediating analyses revealed that: (1) three dimensions of essentialism, challenge, and child-centered within intensive parenting attitudes negatively predict preschoolers’ social-emotional competence, while the two dimensions of fulfillment and stimulation within intensive parenting attitudes positively predict preschoolers’ social-emotional competence; (2) family functioning and psychological resilience mediated the link between intensive parenting attitudes and social-emotional competence, respectively; and (3) family functioning and psychological resilience acted as a sequential mediating chain linking intensive parenting to preschoolers’ social-emotional competence.

**Discussion:**

These findings highlight that parents should appropriately adjust the intensity of their parenting to foster a supportive family environment for preschoolers’ social-emotional growth and nurture their psychological resilience; teachers and related mental health workers should work in synergy with families in preschoolers’ social-emotional competence, and prioritize family-oriented nurturing and intervention measures.

## 1 Introduction

Recently, numerous studies have focused on exploring potential associations between children’s non-cognitive traits and their individual health development and well-being in adulthood, with social–emotional competence receiving much attention as an important topic (Jones et al., 2015). Social–emotional competence refers to the ability to regulate emotions, build positive relationships, and make responsible social decisions to address challenges (Collaborative for Academic, Social, and Emotional Learning, 2017). By four to five years of age, children begin to identify and interpret increasingly complex emotional states (Fernández-Castillo, 2020). The growth of social–emotional competence positively predicts preschoolers’ later academic success, social skills (Krisztián and Barrett, 2018), as well as future career development, and income (Huang, 2024).

Scholars have focused on the important role of parenting knowledge (Zhang, 2018), and parenting styles in influencing preschoolers’ social–emotional competence (Harris et al., 2022). Among these family-related variables, one of the subjects that has attracted considerable attention in recent years is intensive parenting, which was first introduced by Hays (1986) and refers to a parenting style that is child-centered, follows the recommendations of expert guidance, is highly emotionally involved, labor-intensive, economically costly, focuses on stimulating child development, and does the best it can for the child (Hays, 1986; Gauthier et al., 2021). Intensive parenting has become the dominant ideal model of child-rearing across social strata (Ishizuka, 2019). The majority of Chinese parents have begun to deeply engage in their children’s lives through various means such as educational expenditure and time investment, exhibiting characteristics of intensive parenting (Li and Zhang, 2020). Influenced by Confucian educational traditions and the cultural mindset of “expecting children to become dragons,” intensive parenting in China primarily revolves around children’s academic achievements. This manifests as high educational expectations, deep educational intervention, and intensive investment in extracurricular educational resources (Bray, 2009; Wang, 2023). In recent years, with the marketization of education and the proliferation of “shadow education,” the role of mothers has increasingly been constructed as “educational agents,” further intensifying the trend toward more intensive parenting practices (Yang, 2018). A qualitative study conducted against the backdrop of China’s three-child policy reveals that Chinese families exhibit intensive parenting characteristics centered around children in their leisure arrangements, involving substantial financial investment, significant time commitment, and considerable energy expenditure. This pattern suppresses parents’ willingness to have additional children (Shen and Luo, 2023). However, empirical findings concerning the role of intensive parenting in children’s social–emotional development remain mixed. There is a long history of research indicating that parental involvement has been associated with better social and emotional outcomes in children (Nokali et al., 2010; Schiffrin et al., 2015). In contrast, relevant research has shown that there is little correlation between parents engaging preschoolers in time-intensive and costly cognitive and motor activities and increased well-being; because involving children in structured activities reduces their chances for unstructured play with parents or peers, which is known to predict positive outcomes for both children and parents (Schiffrin et al., 2015). These contradictory phenomena indicate that, on the one hand, intensive parenting encompasses complex conceptual dimensions; on the other hand, the advantages of intensive parenting may be conditional—that is, its impact on children’s social–emotional competence may be shaped by other contextual or individual factors. Therefore, it’s important to investigate how these parenting styles relate to preschoolers’ social–emotional competence and the mechanisms involved.

This study is grounded in social ecological theory (Bronfenbrenner, 1979/2010), which posits that development is a “composite function of the individual and the environment.” This theory provides an overarching framework for understanding how children’s development is shaped through the dynamic interaction between the individual and their surrounding environment. Within this framework, the family as a microsystem directly predicts children’s behavioral and emotional development, with child development stemming from sustained interaction within multiple ecological contexts involving the individual, parents, and the family environment. Building upon the theoretical foundations of social ecological theory, this study further incorporates complementary perspectives from family systems theory and psychological resilience—specifically the concept of resource conservation—to achieve a more comprehensive understanding of children’s social–emotional development. Integrating these viewpoints enables multi-level analysis to capture the underlying mechanisms influencing development. Adhering to this methodological framework, the research focuses on examining the relationship between intensive parenting attitudes and preschool children’s social–emotional competencies, with particular attention to the mediating roles of family functioning and psychological resilience.

## 2 Theoretical foundation and research hypotheses

### 2.1 The intensive parenting attitudes and preschoolers’ social–emotional competence

Intensive parenting, as a multidimensional construct, is both a cultural ideology and a concrete parenting practice model. As an ideology, it represents a child-centered, highly invested, expert-guided “good parenting” cultural norm that has become the mainstream ideal model of child-rearing across different social strata. As a parenting practice, it manifests as parents’ intensive investment of time, money, and emotional energy (Li and Huang, 2025). This dual nature implies that the prediction of intensive parenting on child development is not unidimensional. When internalized as cultural beliefs by parents, its various dimensions may produce differentiated effects through distinct psychological mechanisms. Specifically, the five dimensions of intensive parenting attitudes proposed by Liss et al. (2013) manifest distinct behavioral tendencies in parenting practices and exert varying influences on early childhood development. The dimensions of *essentialism*—which posits that women are inherently more adept at childcare than men and should bear the primary responsibility for nurturing—and *challenging*—which characterizes parenting as inherently difficult and the most demanding job—are more closely associated with beliefs about gender roles and perceptions of parenting stress. These dimensions serve as negative predictors of parenting behaviors. Previous studies have suggested that essentialism and challenging, as dimensions of intensive parenting attitudes, have adverse effects on parenting practices. Specifically, essentialism significantly predicts negative parental emotions, including anger, anxiety, shame, and depression (Long and Prikhidko, 2024). Meanwhile, challenging is related to higher levels of negative parenting behaviors, such as inconsistency and harsh discipline (Egami, 2020). In contrast, the *fulfillment* dimension—which asserts that parenting should be deeply fulfilling and joyful—correlates positively with parenting efficacy and positive emotions. This dimension tends to promote adaptive parenting behaviors and positively predicts child development outcomes. The dimensions of *child-centeredness*—which holds that parents should prioritize children’s needs above all else—and stimulation—which advocates that parents should actively stimulate children’s cognitive and intellectual development—exhibit dual effects. On one hand, these dimensions may encourage parents to provide rich cognitive stimulation and emotional support. On the other hand, if pursued beyond reasonable limits, they may evolve into overinvolvement or excessive control, thereby restricting the development of children’s autonomy (Egami, 2024).

### 2.2 The mediating role of family functioning

Family functioning is a composite variable describing the emotional bonds and problem-solving skills of family members (Sell et al., 2021). Family systems theory emphasizes that the family environment subsystem influences children’s socialization. Family functioning, serving as a measure of the operational quality of the entire family ecosystem, functions both as a functional variable within the family ecosystem and as a significant mediating variable (Olson, 2000; Guo, 2023). Therefore, the interaction between parenting styles and family functioning affects children’s psychological development. On the one hand, parenting styles could profoundly predict family functioning (Zhang et al., 2017); positive parenting styles increase emotional ties among family members, which is conducive to the establishment of family functioning (Sun and Xu, 2019). On the other hand, the research indicates that when the family fails to perform its basic functions effectively, family members may have various clinical problems (Miller et al., 2010), and on the contrary, positive and harmonious family relationships are conducive to the establishment of secure attachment relationships between preschoolers and their parents, and strengthen their emotional security (Wang et al., 2021). Relevant empirical studies have shown that good communication and emotional responsiveness in family functioning have significant protective effects on preschoolers’ autistic behaviors (Xie et al., 2024), and emotional response and behavioral control have a negative predictive effect on preschoolers’ social anxiety (Liu et al., 2020).

### 2.3 The mediating role of psychological resilience

Psychological resilience is the ability to recover from adversity and adapt to changing circumstances (Peng et al., 2022). A positive family environment can satisfy the psychological needs of preschoolers to gain a sense of security and love, which promotes the natural formation of psychological resilience, protects them from risk factors, and reduces the emergence of emotional-behavioral problems (Xing et al., 2016). On the one hand, family parenting styles shape the development of psychological resilience in preschoolers. This process involves both risk and protective factors: supportive and warm parenting can foster resilience, while rejecting and overprotective styles may hinder it (Zeng and Li, 2003; Du et al., 2021). Family parenting styles in intensive parenting are characterized by both authoritative and authoritarian characteristics (Li and Wen, 2021), and there is a possibility of negatively affecting psychological resilience in preschoolers. Empirical studies have also shown that authoritarian parenting attitudes and practices and overprotective parenting attitudes and practices are negatively associated with children’s psychological resilience (Guo et al., 2016; Sun and Xu, 2023; Kılıçoğlu et al., 2024). On the other hand, the development of psychological resilience in preschoolers also affects their social–emotional competence. Besides, psychological resilience could predict children’s well-being (Zhu et al., 2018), and that emotional-behavioral problems can be improved by increasing their psychological resilience (Ban et al., 2022). Meanwhile, higher psychological resilience is associated with fewer emotional behavior problems (Ban et al., 2022; Benzies and Mychasiuk, 2010).

Meanwhile, the dynamic model of psychological resilience states that when family functioning as an external resource is achieved, it can naturally promote the development of internal resources such as children’s psychological resilience (Li and Zhang, 2006; Wang et al., 2020). Family functioning can predict psychological resilience. Empirical studies have found that good family functioning strengthens the psychological resilience of disadvantaged children. (Lv and Zhou, 2023); for children in general situations, cumulative family risk has a negative effect on their psychological resilience (Liu et al., 2023).

### 2.4 The present study

Although extensive research has explored the influencing factors and internal mechanisms of children’s social–emotional competencies, dedicated studies on the pre-school stage remain insufficient in both scope and depth. Most prior research has focused on either intensive parenting practices or intensive parenting attitudes as single dimensions, failing to adequately examine the broader ecological interactions and underlying mechanisms between families and individuals. Moreover, few studies have integrated micro-level contextual factors (e.g., family functioning) and intrinsic mechanisms (e.g., psychological resilience) within a unified analytical framework.

To fill this gap, the present study utilizes a large, multi-province sample of typically developing preschool children in China to examine how five dimensions of intensive parenting attitudes relate to children’s social–emotional competencies through family functioning and psychological resilience. The chained mediation framework proposed here transcends existing research by simultaneously capturing both micro-situational factors within the family and psychological internal mechanisms, thereby offering a more comprehensive explanation of preschool children’s social–emotional development within the context of socio-educational environment.

Building on these aims, the present study focuses on investigating how intensive parenting attitudes relate to the social–emotional competencies of preschool children, with particular attention to the chain-mediated role of family functioning and psychological resilience.

Based on the stated research aims and theoretical framework, the following hypotheses are proposed:

*H1*: Various types of intensive parenting styles have predictive effects on preschoolers’ social–emotional competence.

*H2*: Family functioning mediates the association between various types of intensive parenting attitudes and preschoolers’ social–emotional competence.

*H3*: Psychological resilience mediates the association between various types of intensive parenting attitudes and preschoolers’ social–emotional competence.

*H4*: Family functioning and psychological resilience play a chain mediating role in the association of various types of intensive parenting attitudes on preschoolers' social-emotional competence.

## 3 Methods

### 3.1 Participants

A convenience sampling method was used to select kindergarten preschoolers in four provinces of China as participants. Because preschoolers are unable to complete self-report questionnaires accurately, their parents who possess comprehensive knowledge of their children’s daily behaviors and emotional states were invited to complete the online survey on behalf of their children during December 2024. A total of 2,250 questionnaires were distributed, with 2,174 valid responses collected, yielding a 96.6% response rate. To control for the potential influence of demographic factors on the study variables, this research collected information on the children’s gender, family structure, primary caregiver, family residence, and age. Among them, gender was categorized as boys and girls, with 1,118 (51.4%) and 1,156 (48.6%) respectively; Family structure was divided into only children and non-only children, with 599 (27.6%) and 1,575 (72.4%) respectively; Primary caregivers were categorized into single parent, both parents, and other types, comprising 749 individuals (34.5%), 1,177 individuals (54.1%), and 248 individuals (11.4%), respectively; Family residence was divided into urban and rural areas, with 1,669 individuals (76.8%) and 505 individuals (23.2%), respectively. The age of the preschool children ranged from 3 to 6 years (*M* = 4.72, *SD* = 0.877).

### 3.2 Measures

#### 3.2.1 Intensive parenting attitude questionnaire

The Intensive Parenting Attitudes Questionnaire (Liss et al., 2013) was used. The questionnaire comprises five dimensions: essentialism, fulfillment, stimulation, challenge, and child-centered, totaling 25 items. The essentialism dimension includes 7 items (e.g., “Both fathers and mothers are equally capable of caring for children”). The fulfillment dimension includes 4 items (e.g., “Parenting is not the most rewarding thing a person can do”). The stimulation dimension includes 4 items (e.g., “It is important for children to participate in classes, lessons, and activities that engage and stimulate them”). The challenge dimension comprises 6 items (e.g., “Parenting is exhausting”). The child-centered dimension includes 3 items (e.g., “Children should be the center of attention”). The questionnaire used a six-point Likert scale, ranging from 1 (“strongly disagree”) to 6 (“completely agree”). In the scale, items 1, 10, and 16 are reverse-scored. The average score for all questions was calculated by reverse scoring some of the questions in the scale, with higher scores indicating a more far-reaching and intensive parenting style. The Cronbach’s alpha of the scale in this study was 0.7 and each dimension was 0.67, 0.61, 0.56, 0.62, and 0.56, respectively. The Cronbach’s *α* coefficients for each subscale ranged from 0.5 to 0.7. This result aligns with measurement data for these dimensions in Chinese and Japanese samples (Li et al., 2024; Egami, 2024), indicating that their internal consistency reliability falls within an acceptable range. The goodness of fit index gave the following values to test the construct validity of the scale: χ^2^/df = 8.99^***^, RMSEA = 0.06, CFI = 0.84, TLI = 0.81, SMAR = 0.08. Although the CFI and TLI values are slightly below the recommended 0.90 threshold, both the RMSEA and SRMR values meet the stringent fit criteria of 0.06 and 0.08 (Hu and Bentler, 1999). From a methodological perspective, the evaluation of model fit should be based on a comprehensive assessment of multiple indices (Wen et al., 2004; Khademi et al., 2023). Furthermore, given that the application of this parenting attitude scale within the Chinese cultural context remains in the exploratory research phase (Li et al., 2024), fit criteria may be interpreted with moderate flexibility. Considering all fit indices collectively, the model demonstrates acceptable goodness-of-fit in this study.

#### 3.2.2 Social–emotional competence scale

The Chinese Inventory of Children’s Social–emotional Competence (Li et al., 2020) was used. The scale contains 30 items (e.g., “Tends to forget longer instructions.” “Takes initiative to care for others.”). The questionnaire employed a five-point Likert scale, ranging from 1 (“strongly disagree”) to 5 (“strongly agree”). In the scale, items 1–13 and 25–30 are reverse-scored. The mean score of all the questions was calculated by reverse scoring some of the questions in the scale. Higher scores reflected greater social–emotional competence in children. The Cronbach’s alpha of the scale was 0.91. The goodness of fit index gave the following values to test the construct validity of the scale: χ^2^/df = 9.88^***^, RMSEA = 0.06, CFI = 0.88, TLI = 0.87, SMAR = 0.05. The measurement model fit indices are acceptable.

#### 3.2.3 Family functioning scale

The Family Functioning Assessment Scale (FFAS) was used. The scale was developed by Epstein et al. (1983). This study employed the Short Form General Functioning Scale, adapted into Chinese by Li (2021), which has been demonstrated in prior research as a rapid assessment tool for family functioning (Xie et al., 2024). The scale contains 12 items (e.g., “It is difficult to arrange some family activities because we misunderstand each other.” “We are able to support each other in times of crisis.”). The questionnaire adopted a four-point Likert scale, ranging from 1 (“very much like my family”) to 4 (“not at all like my family”). In the scale, items 1, 3, 5, 7, 9, and 11 are reverse-scored. In the original questionnaire, a higher score indicates poorer family functioning. In this study, to maintain consistency with the logic of other scales, the overall scale scores were reverse-scored in accordance with existing research (Liu et al., 2020). Higher total scores indicate better family functioning. The Cronbach’s alpha of the scale in this study was 0.84. The goodness of fit index gave the following values to test the construct validity of the scale: χ^2^/df = 19.54^***^, RMSEA = 0.09, CFI = 0.92, TLI = 0.86, SMAR = 0.07. The measurement model exhibits good fit metrics.

#### 3.2.4 Psychological resilience scale

Psychological resilience was measured using the second edition of the Devereux Early Childhood Assessment for Preschoolers. The scale was developed by LeBuffe and Naglieri (2012) and revised by Ji et al. (2015). The scale contains 27 items (e.g., “My child behaves in a way that makes adults laugh or show interest in him/her.” “My child listens to or respects others.”). The questionnaire employed a five-point Likert scale, ranging from 0 (“not at all”) to 4 (“completely”). The higher the score, the higher the level of psychological resilience. The Cronbach’s alpha of the scale was 0.94. The goodness of fit index gave the following values to test the construct validity of the scale: χ^2^/df = 9.63^***^, RMSEA = 0.06, CFI = 0.91, TLI = 0.89, SMAR = 0.05. The measurement model exhibits good fit metrics.

### 3.3 Data processing

The data analysis proceeded in three stages. First, after data entry into SPSS 23.0, an unrotated exploratory factor analysis was conducted to perform Harman’s single-factor test, which assessed common method bias by examining whether a single factor accounted for the majority of variance among all measurement items. Second, Pearson correlation analyses and comparative analyses were conducted to examine the associations and group differences among the primary study variables, providing preliminary insights into their interrelationships and demographic variations. In the analysis, variables such as gender, only-child status, primary caregiver, and household residence were converted into dummy variables and incorporated into the model. Third, verification factor analysis, structural equation modeling, and mediation analysis were conducted using Mplus 8.3.

## 4 Results

### 4.1 Common methodological biases

In order to avoid common method bias, this study included measures such as anonymous responses and reverse-coded items. To further ensure rigor, the Harman single-factor test was used, revealing 18 factors with eigenvalues above 1; the first factor explained only 19.22% of the variance, below the 40% threshold. This suggests that serious common method bias was not present in the data. This study employed parent-reported measures to collect data on parenting attitudes and child development. Although single-source information may be subject to common method bias, parent-reported measures have been widely validated as effective and reliable instruments in child development research (Carey, 1982; Waldman et al., 2025). As primary caregivers in children’s daily lives, parents possess the most direct and continuous observations of children’s behavioral patterns and developmental status, providing ecological validity that is difficult for other information sources to replicate (Wu et al., 2025).

### 4.2 Descriptive statistics and correlation analysis

The correlation analyses are presented in Table 1. The three dimensions of essentialism, challenge, and Child-Centred within intensive parenting attitudes were found to be significantly negatively correlated with the three core variables of family functioning, psychological resilience, and social–emotional competence. The two dimensions of fulfillment and stimulation within intensive parenting attitudes were found to be significantly positively correlated with all three core variables of family functioning, psychological resilience, and social–emotional competence. Family functioning is significantly positively correlated with psychological resilience and social–emotional competence, while psychological resilience is markedly positively correlated with social–emotional competence. Age was found to correlate with key variables including fulfillment, family functioning, and social–emotional competence. Consequently, subsequent research incorporated age as a control variable within the model.

**Table 1:** Correlation results.

Variables	*M*	*SD*	1	2	3	4	5	6	7	8	9
1. Age	4.72	0.88	1								
2. Essentialism	3.08	0.75	0.16	1							
3. Fulfillment	4.70	0.76	−0.48^*^	−0.24^***^	1						
4. Stimulation	4.79	0.66	−0.32	−0.15^***^	0.41^***^	1					
5. Challenge	4.05	0.74	−0.39	0.36^***^	−0.02	0.19^***^	1				
6. Child-centered	3.70	0.91	0.00	0.25^***^	0.16^***^	0.15^***^	0.31^***^	1			
7. Family functioning	3.03	0.41	−0.06^**^	−0.48^***^	0.28^***^	0.25^***^	−0.25^***^	−0.18^***^	1		
8. Psychological resilience	1.97	0.41	0.02	−0.37^***^	0.26^***^	0.20^***^	−0.24^***^	−0.13^***^	0.45^***^	1	
9. Social–emotional competence	3.58	0.50	0.07^**^	−0.36^***^	0.26^***^	0.18^***^	−0.30^***^	−0.16^***^	0.44^***^	0.51^***^	1

^*^p < 0.05, ^**^p < 0.01, ^***^p < 0.001, Boys = 1 Girls = 2, Only child = 1 Non-only child = 2, Primary caregiver mother = 1 Father = 2 Parents together = 3 Grandparents = 4 Other = 5, Rural = 1 Urban = 2.

### 4.3 Comparative analysis

After presenting the overall descriptive statistics and correlations among the key study variables, group comparisons were conducted to examine whether key variables differed by gender, only-child status, primary caregiver and family residence. The results of these comparative analyses are summarized in Table 2.

**Table 2:** Differential analysis of demographic variables.

Variables	Essentialism	Fulfillment	Stimulation	Challenge	Child-centered	Family functioning	Psychological resilience	Social–emotional competence
Gender								
Boy (*n* = 1,118)	3.07 ± 0.75	4.68 ± 0.75	4.79 ± 0.66	4.03 ± 0.75	3.69 ± 0.90	3.01 ± 0.40	3.85 ± 0.47	3.53 ± 0.48
Girl (*n* = 1,056)	3.08 ± 0.76	4.73 ± 0.76	4.80 ± 0.65	4.06 ± 0.75	3.71 ± 0.91	3.05 ± 0.42	3.90 ± 0.51	3.63 ± 0.51
*t*	−0.36	−1.46	−0.52	−0.90	−0.49	−1.94	−2.44^*^	−4.53^***^
Only-child status								
Only child (*n* = 599)	3.01 ± 0.79	4.67 ± 0.79	4.84 ± 0.65	3.98 ± 0.80	3.58 ± 0.94	3.09 ± 0.41	3.87 ± 0.48	3.57 ± 0.49
Non-only child (*n* = 1,575)	3.10 ± 0.74	4.72 ± 0.74	4.78 ± 0.66	4.07 ± 0.73	3.74 ± 0.89	3.00 ± 0.40	3.87 ± 0.49	3.58 ± 0.50
*t*	−2.49^*^	−1.53	−1.89	−2.67^**^	−3.89^***^	4.51^***^	0.55	−0.60
Primary caregiver								
Single parent (*n* = 749)	3.21 ± 0.73	4.62 ± 0.74	4.75 ± 0.64	4.08 ± 0.76	3.64 ± 0.88	2.97 ± 0.38	3.84 ± 0.47	3.54 ± 0.49
Both parents (*n* = 1,177)	2.99 ± 0.75	4.76 ± 0.76	4.81 ± 0.67	4.02 ± 0.75	3.71 ± 0.92	3.07 ± 0.42	3.91 ± 0.50	3.63 ± 0.49
others (*n* = 248)	3.08 ± 0.77	4.71 ± 0.77	4.85 ± 0.65	4.08 ± 0.72	3.83 ± 0.88	3.01 ± 0.42	3.80 ± 0.50	3.45 ± 0.51
*F*	18.62^***^	8.09^***^	3.42^*^	2.31	4.08^*^	16.62^***^	7.18^**^	16.59^***^
Residential location								
Rural (*n* = 505)	3.19 ± 0.74	4.63 ± 0.71	4.71 ± 0.68	4.13 ± 0.75	3.87 ± 0.82	2.92 ± 0.37	3.75 ± 0.54	3.48 ± 0.48
Urban (*n* = 1,669)	3.04 ± 0.75	4.72 ± 0.77	4.82 ± 0.64	4.02 ± 0.74	3.65 ± 0.92	3.06 ± 0.41	3.91 ± 0.47	3.61 ± 0.50
*t*	3.96^***^	−2.40^*^	−3.38^**^	2.79^**^	5.16^***^	−7.34^***^	−6.02^***^	−5.31^***^

^*^p < 0.05, ^**^p < 0.01, ^***^p < 0.001.

Scores on the five dimensions of intensive parenting attitudes showed significant differences across only-child status, primary caregivers, and residential backgrounds, but no significant differences based on child gender. Family functioning scores showed significant differences across only-child status, primary caregiver status, and residential background, but no significant differences based on child gender. Psychological resilience scores showed significant differences across gender, primary caregiver status, and residential background, but no significant differences in only-child status. Social–emotional competency scores showed significant differences across gender, primary caregiver status, and residential background, but no significant differences in only-child status.

Accordingly, gender, only-child status, primary caregiver status and residential background were included as control variables in subsequent analyses to account for their potential predictive role on the primary relationships among intensive parenting attitudes, family functioning, psychological resilience, and social–emotional competence.

### 4.4 Analysis of mediating role

**Figure 1:**
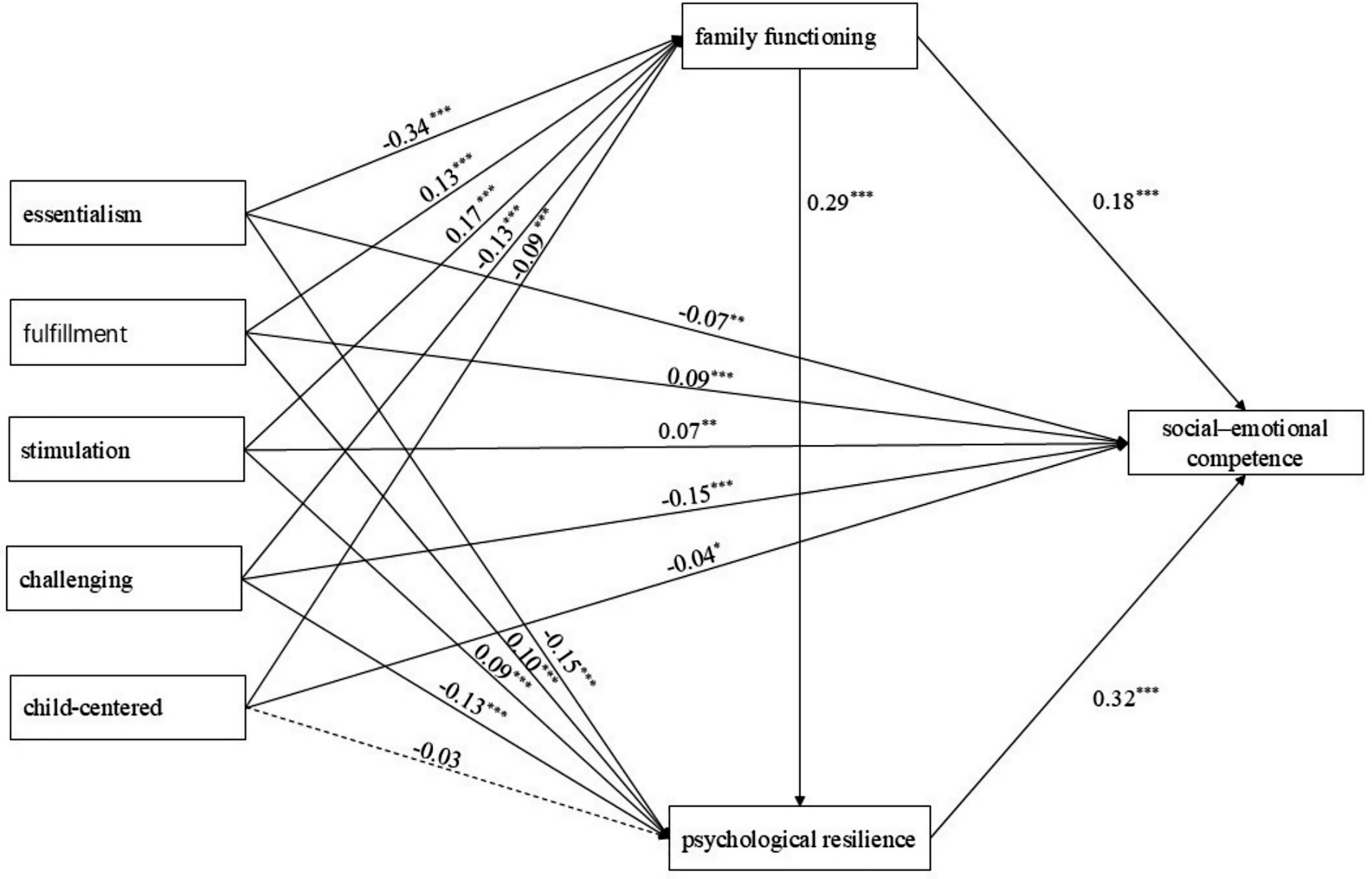
Mediating role of family functioning and psychological resilience in intensive parenting attitude on social–emotional competence.

Building upon the comparative analysis, this study employed Mplus 8.3 to construct a structural equation model analyzing the relationships among five dimensions of intensive parenting attitudes, family functioning, psychological resilience, and preschoolers’ social–emotional competencies. The study controlled for family structure, primary caregiver characteristics, residential background, child gender, and age. The model structure is depicted in Figure 1. Model fit results indicate a saturated model (CFI = 1.000, TLI = 1.000).

Essentialism (*β* = −0.34, *p* < 0.001), challenging (*β* = −0.13, *p* < 0.001), and child-centered (*β* = −0.09, *p* < 0.001) within intensive parenting attitudes significantly negatively predicted family functioning. Fulfillment (*β* = 0.13, *p* < 0.001) and stimulation (*β* = 0.17, *p* < 0.001) significantly and positively predicted family functioning. Essentialism (*β* = −0.15, *p* < 0.001) and challenging (*β* = −0.13, *p* < 0.001) within intensive parenting attitudes significantly negatively predicted psychological resilience. Fulfillment (*β* = 0.10, *p* < 0.001) and stimulation (*β* = 0.09, *p* < 0.001) significantly and positively predicted psychological resilience, whereas the child-centered dimension showed no significant predictive effect. Essentialism (*β* = −0.07, *p* < 0.01), challenging (*β* = −0.15, *p* < 0.001), and child-centered (*β* = −0.04, *p* < 0.05) within intensive parenting attitudes significantly negatively predicted social–emotional competence. Fulfillment (*β* = 0.09, *p* < 0.001) and stimulation (*β* = 0.07, *p* < 0.01) significantly and positively predicted social–emotional competence. Family functioning significantly and positively predicted psychological resilience (*β* = 0.29, *p* < 0.001) and social–emotional competence (*β* = 0.18, *p* < 0.001). Psychological resilience significantly and positively predicted social–emotional competence (*β* = 0.32, *p* < 0.001).

The bootstrap method with bias correction (5,000 resamples, 95% confidence interval) was employed to examine the mediating effects of family functioning and psychological resilience, as shown in Table 3. Family functioning partially mediates the relationship between the five dimensions of intensive parenting attitudes and social–emotional competence, with the 95% confidence interval not containing 0. Psychological resilience partially mediates the relationship between intensive parenting attitudes—specifically in the dimensions of essentialism, challenging, fulfillment, and stimulation—and social–emotional competence, with the 95% confidence interval not containing 0. Family functioning-psychological resilience exerts a chain-mediated effect between five dimensions of intensive parenting and social–emotional competence, with the 95% confidence interval not including 0.

**Table 3:** Chained mediation role test.

Independent variable	Path	*β*	95%CI	
			Lower	Upper
Essentialism	essentialism → family functioning → social–emotional competence	−0.06	−0.079	−0.044
	essentialism → psychological resilience → social–emotional competence	−0.05	−0.062	−0.031
	essentialism → family functioning → psychological resilience → social–emotional competence	−0.03	−0.040	−0.024
Fulfillment	fulfillment → family functioning → social–emotional competence	0.02	0.013	0.034
	fulfillment → psychological resilience → social–emotional competence	0.03	0.019	0.047
	fulfillment → family functioning → psychological resilience → social–emotional competence	0.01	0.007	0.018
Stimulation	stimulation → family functioning → social–emotional competence	0.03	0.020	0.042
	stimulation → psychological resilience → social–emotional competence	0.03	0.015	0.045
	stimulation → family functioning → psychological resilience → social–emotional competence	0.02	0.011	0.022
Challenge	challenge → family functioning → social–emotional competence	−0.02	−0.034	−0.013
	challenge → psychological resilience → social–emotional competence	−0.04	−0.054	−0.026
	challenge → family functioning → psychological resilience → social–emotional competence	−0.01	−0.017	−0.007
Child-centered	child-centered → family functioning → social–emotional competence	−0.02	−0.025	−0.008
	child-centered → psychological resilience → social–emotional competence	−0.01	−0.021	0.005
	child-centered → family functioning → psychological resilience → social–emotional competence	−0.01	−0.013	−0.004

## 5 Discussion

This study, centered on preschoolers’ social–emotional competence, reveals the predictive role of five dimensions of intensive parenting attitudes on social–emotional competence and verifies the chain-mediating roles of family functioning and psychological resilience. It demonstrates that the three dimensions of essentialism, challenge, and child-centered within intensive parenting attitudes not only directly undermine children’s emotional and social development but also indirectly predict it through strained family dynamics and reduced psychological resilience. These findings provide a clearer understanding of the risks associated with high-pressure parenting and offer both theoretical support and practical recommendations for promoting healthier parenting strategies and supporting children’s emotional well-being in early childhood (Table 3).

### 5.1 Associations between intensive attitudes and preschoolers’ social–emotional competence

Intrusive parenting reflects excessive control over a child’s behavior or psychological state, overlapping conceptually with intensive parenting. Recent studies indicate that intrusive parenting is associated with internalizing and externalizing symptoms in adolescents. Among these, the strongest relationship is observed with the subtypes of parental psychological controlling behavior and overprotective parenting, while the association with helicopter parenting is relatively weaker (Ryan et al., 2026). This finding suggests that different types of “high-intensity” parenting yield heterogeneous psychological consequences. The intensive parenting attitudes examined in this study encompass dimensions that may lead to psychological control (e.g., “essentialism” and “challenging behavior”) as well as dimensions that may promote positive engagement (e.g., “sense of accomplishment”), providing a theoretical foundation for its dual positive and negative predictions.

Results showed that the three dimensions of essentialism, challenging, and child-centeredness within intensive parenting attitudes negatively predict preschoolers’ social–emotional development, which is consistent with prior research findings (Egami, 2024). First, regarding essentialism, this dimension often imposes psychological and economic pressures on mothers (Li and Wen, 2021; Li et al., 2024), which in turn significantly predicts young children’s social–emotional development. Secondly, regarding the dimension of challenging, the stress experienced by parents may predict their parenting behaviors, thereby deteriorating the quality of parent–child relationships and ultimately hindering young children’s social–emotional development (Mefharet, 2023). Economic stress is associated with parental emotional distress, which in turn reduces the quality of family learning activities through negative parent–child interactions, thereby hindering young children’s social–emotional development (Li et al., 2023). Finally, regarding the child-centered dimension, this perspective may implicitly reflect a view that children are vulnerable and lacking in agency (Yerkes et al., 2021), while also implying strict discipline (Egami, 2024). Overly intrusive parental behavior may place children in a state of vulnerability and passivity, undermining the development of their autonomy and innovative abilities, and negatively predicts the development of their social–emotional skills. Related research also indicates that excessive parental involvement increases children’s risk of depression in adulthood and reduces their life satisfaction (Schiffrin et al., 2014).

The two dimensions of fulfillment and stimulation within intensive parenting positively predict young children’s social–emotional competencies, consistent with prior research findings (Liss et al., 2013; Egami, 2024). Parental efficacy and enjoyment of parenting lead to positive parenting behaviors. “Stimulating children’s development” shows a low positive correlation with preschoolers’ subjective well-being (Schiffrin et al., 2015). This may stem from the fact that parents’ unconditional positive regard and appropriate assistance create a favorable psychological environment for children’s development (Deng et al., 2024).

### 5.2 The mediating role of family functioning

The present study found that positive elements of intensive parenting attitudes (e.g., fulfillment and stimulation) predict the development of good family functioning, while negative elements within intensive parenting attitudes (e.g., essentialism, challenging, and child-centered) predict the development of poor family functioning, consistent with previous research (Zhang et al., 2017; Zhang et al., 2018; Carlo et al., 2007). Meanwhile good family functioning positively predicts preschoolers’ social–emotional competence, consistent with previous studies (Long and Prikhidko, 2024; Egami, 2024; Yi, 2022). Thus, the mediating role of family functioning holds. On the one hand, the negative elements within intensive parenting attitudes may lead to poor family functioning. Family functioning, as a combination of individual interaction styles, role assignments, and interpersonal relationship patterns and operations within the family system, is significantly constrained and influenced by the quality of family relationships in terms of its overall efficacy. One explanation is that negative parenting styles, such as overprotection, tend to make parent–child relationships distant and parent–child communication blocked, resulting in a poor family atmosphere and affecting family functioning (Huang et al., 2021). Another explanation suggests that negative elements within intensive parenting attitudes may impose stress on families and negatively predict family functioning. Parents may feel a sense of failure when the desired parenting pattern is not achieved; Excessive investment of time, energy, and money in children may be linked to the psychological and financial pressures parents endure (Li and Wen, 2021; Li et al., 2024). The five-dimensional prediction pathways of intensive parenting attitudes on family functioning can be understood within the broader context of helicopter parenting research. Helicopter parenting exhibits dual characteristics of control and care (Padilla-Walker and Nelson, 2012). Empirical research supports this perspective, with Wang and Hawk (2023) finding that in Chinese parent–child relationships, helicopter parenting both negatively and positively correlates with the quality of child–mother relationships. One possible explanation is that children tend to rationalize their parents’ overbearing behavior. When perceived helicopter parenting exceeds normal levels, children may feel constrained, leading to conflict with their parents. However, after the conflict subsides, they may defend their parents’ actions, interpreting them as expressions of care rather than control, ultimately attributing parental intervention to nurturing rather than interference (Wang et al., 2025).

Additionally, poor family functioning affects preschoolers’ social–emotional competence development, a finding that supports the family functioning model (Beavers, 1981) and family systems theory (Cox and Paley, 2003). Family functioning is related to the interaction of multiple subsystems in the family. When family dysfunction exists, multiple subsystems, such as marital relationships and parent–child relationships, may change in an unfavorable direction and interact dynamically, which negatively predicts children’s psychological health (Wen et al., 2024). Empirical studies have also pointed out that poor family functioning is associated with child anxiety. Poor family functioning may create barriers to effective parent–child communication, leading to difficulties for children in obtaining adequate emotional support and understanding. Due to the lack of open channels of communication and emotional fulfillment, children are unable to adequately express their inner experiences, which further exacerbates the accumulation of anxiety (Huang et al., 2024).

### 5.3 The mediating role of psychological resilience

We also found that positive elements of intensive parenting attitudes (e.g., fulfillment and stimulation) positively predict the development of psychological resilience, while negative elements within intensive parenting attitudes (e.g., essentialism and challenging) negatively predict the development of psychological resilience, consistent with previous research, consistent with previous studies (Guo et al., 2016; Sun and Xu, 2023; Kılıçoğlu et al., 2024), Meanwhile psychological resilience positively predicts preschoolers’ social–emotional competence, consistent with previous studies (Zhu et al., 2018; Ban et al., 2022). This result is consistent with the theory of resource preservation in psychological resilience research (Tian and Liu, 2024). The fulfillment of children’s developmental needs for love, belonging, and safety relies on external resources such as the family. If external resources provide fulfillment of children’s psychological needs, children will develop internal resources such as psychological resilience and promote their healthy growth. On the one hand, the negative elements within intensive parenting attitudes adversely predict the development of psychological resilience in preschoolers. Relevant studies have demonstrated that authoritarian parenting and overprotection negatively predict children’s psychological resilience (Guo et al., 2016; Jin and Liu, 2018). In contrast, lowering parental psychological control can effectively foster the development of preschoolers’ intrinsic motivation, thereby encouraging their active engagement in cognitive exploration activities (Townshend, 2016), and ultimately enhance their psychological resilience level. Besides, preschoolers’ psychological resilience supports the development of social–emotional competence. When faced with environmental stress or challenges, preschoolers display various coping patterns. Children with higher psychological resilience are more inclined to adopt proactive environmental adjustment strategies, such as positively regulating their negative emotional and behavioral responses and strengthening parent–child attachment. Such adaptive coping styles significantly reduce the probability of internalizing problem behaviors (Ye et al., 2023).

Additionally, the study found that family functioning and psychological resilience jointly formed a chain-mediated pathway between intensive parenting and social–emotional competence. Related studies have also examined and confirmed the chain mediating role of family functioning and personal psychological characteristics (self-esteem) in family parenting styles and children’s psychological cognition and behaviors (life satisfaction, self-control) (Hou et al., 2018; Guo, 2023). Family functioning predicts preschoolers’ social–emotional competence both directly and indirectly through psychological resilience. Good family functioning, as an important external social resource influencing children’s psychological development, plays a key role in promoting the maturity and perfection of their internal psychological mechanisms. Developmental resources refer to relevant experiences, relationships, skills, and values that effectively promote children’s attainment of healthy developmental outcomes, encompassing external resources (e.g., family environment) and internal resources (e.g., psychological resilience) (Benson et al., 1998; Wang et al., 2020). The greater the number of resources children acquire, the better their development. The interaction between family functioning and psychological resilience facilitates children’s access to better external and internal resources, thereby promoting the development of social–emotional competence. In addition, when the family functions poorly, family relationships are distant or even tense, family intimacy is low, communication between family members is poor, and the frequency and quality of interactions are low. This creates a tense and depressing family atmosphere, in which children’s emotions are also chronically under high levels of stress, resulting in children’s emotional problems (Wang et al., 2021). In this context, psychological resilience serves as a protective factor that can mitigate the negative impact of family dysfunction on the development of preschoolers’ social–emotional competence. Research indicates that psychological resilience can be seen as a broad protective capacity that individuals acquire within themselves and through the many processes of their interactions with the systems in which they live (Wang et al., 2024). When faced with a dysfunctional family, individuals are able to use their psychological resilience to adapt to and manage these external pressures, thereby reducing the risk of problematic behaviors (Mesman et al., 2021).

## 6 Implications, limitations, and suggestions

In terms of theory, this study started from the parenting attitude in the family microsystem to explore their predictive role on preschoolers’ emotional competence, and selected micro-level contextual factors (family functioning) and individual internal characteristics (psychological resilience) for the investigation of internal mechanisms. The study further validates the applicability of the social-ecological system theory, the family systems theory, and the psychological resilience dynamic model in the Chinese preschool population. Research findings indicate that despite statistically significant results, the independent contribution of intensive parenting attitudes to child development is relatively limited. This contribution must be indirectly realized through chained mediating pathways involving family functioning and psychological resilience. In terms of practice, the study reveals that parents of preschoolers need to view intensive parenting correctly and adjust the intensity of parenting in practice at the right time (Li et al., 2024). Meanwhile, a family environment centered on emotional support is constructed to provide a safe and accepting family atmosphere and environment for preschoolers’ social–emotional development. When preschoolers are experiencing difficulties and frustrations, parents should avoid excessive intervention or substitution, pay extra attention to the emotional and behavioral changes of preschoolers and the development of their psychological resilience, stimulate the positivity and initiative of preschoolers to solve problems independently, improve their psychological resilience, and improve their methods and skills of emotional regulation (Huang and Wang, 2024). Moreover, kindergarten teachers and mental health professionals should collaborate closely with families to support the development of preschoolers’ social–emotional competence. Special attention should be given to family functioning factors, prioritizing family-centered interventions and nurturing approaches. In particular, early childhood education institutions should integrate family-based mental health support into their regular programming, offer workshops on positive parenting, and establish communication channels between teachers and caregivers to ensure developmental consistency across settings. This multi-party cooperation can build a holistic support system that promotes children’s emotional well-being in both home and school environments. In family education guidance practices, priority should be given to nurturing beliefs that stimulate positive emotional experiences in parents, rather than solely emphasizing the hardships, challenges, and inherent gender differences in parenting. Overemphasizing the latter not only fails to benefit children’s development but may also exacerbate parental stress and negative emotions.

This study has several limitations due to research conditions. First, cross-sectional studies could not establish causal links between the variables. Parenting is a dynamic process, with parents and their children often influencing each other. Intensive parenting can be both a precursor to poor family relationships and a consequence of them. Subsequent studies should examine the directionality of the variables using a tracer study paradigm. Second, the study only explored the mediating role of family functioning and psychological resilience. In the future, the mediating role of other positive developmental resources and the moderating role of other environmental factors outside the family system could be considered. This will enable a more comprehensive and in-depth understanding of how various factors predict preschoolers’ social–emotional competence, enriching insights into their underlying mechanisms within an ecosystemic framework and the context of collaborative parenting between families and society. Third, although Harman’s single-factor test did not indicate severe common method variance in this study, all variables were collected via parent self-reports. This single-source design may systematically inflate or deflate the observed relationships among variables. Specifically, when parents report both their parenting attitudes and children’s developmental outcomes, they may be influenced by factors such as social desirability, emotional state, and implicit theories. This can lead to the “shared common methods” issue between the predictor and outcome variables (Podsakoff et al., 2003). Furthermore, parents’ reports of children’s behavior often reflect their own psychological characteristics or cognitive biases, making it difficult to fully distinguish between the child’s objective behavior and the parent’s subjective perception. Future research will employ multiple information sources such as teacher evaluations or multi-wave longitudinal designs to more effectively control for and examine common method bias. Fourth, the measurement model employs a saturated model specification, which has its application scenarios during the measurement model phase. However, this approach significantly limits the interpretability of model fit and weakens the argumentation supporting the theoretical structure’s validity. Future research could explore latent variable models or adopt more restrictive model specifications for in-depth investigation.

## 7 Conclusion

The study found that the three dimensions of essentialism, challenge, and child-centered within intensive parenting attitudes negatively predict young children’s social–emotional competencies; the two dimensions of fulfillment and stimulation within intensive parenting attitudes positively predict young children’s social–emotional competencies. Intensive parenting attitudes not only directly predict the development of social–emotional competence but also exert a chain-mediated effect through family functioning and preschoolers’ psychological resilience. This study highlights the risks of intensive parenting in fostering preschoolers’ social–emotional competence and underscores the importance of enhancing family functioning, nurturing preschoolers’ psychological resilience, and fostering collaboration between preschoolers and their families.

## Data Availability

The raw data supporting the conclusions of this article will be made available by the authors, without undue reservation.

## References

[ref1] Ban Y. Sun J. Kong F. (2022). An intervention research of supportive group counseling on resilience and emotional-behavioral problems of street children. Chin. J. Clin. Psychol. 30, 991–995. doi: 10.16128/j.cnki.1005-3611.2022.04.046

[ref2] Beavers R. (1981). “Healthy families,” in Annual Review of Family Therapy, eds. Berenson G. White H. (New York: Human Sciences Press), 57–59.

[ref101] Benson P. L. Leffert N. Scales P. C. Blyth D. A. (1998). Beyond the “village” rhetoric: Creating healthy communities for children and adolescents. Appl. Dev. Sci. 2, 138–159. doi: 10.1207/s1532480xads0203_3

[ref3] Benzies K. Mychasiuk R. (2010). Fostering family resiliency: a review of the key protective factors. Child Fam. Soc. Work 14, 103–114. doi: 10.1111/j.1365-2206.2008.00586.x

[ref4] Bray M. (2009). Confronting the Shadow Education System (Fundamentals of Educational Planning No. 61). Paris, France: UNESCO International Institute for Educational Planning.

[ref5] Bronfenbrenner U. (1979/2010). The Ecology of Human Development. Taipei: Psychological Publishing Co., Ltd.

[ref6] Carey W. B. (1982). Validity of parental assessments of development and behavior. Am. J. Dis. Child. 136, 97–99. doi: 10.1001/archpedi.1982.03970380009001, 7064939

[ref7] Carlo G. McGinley M. Hayes R. Batenhorst C. Wilkinson J. (2007). Parenting styles or practices? Parenting, sympathy, and prosocial behaviors among adolescents. J. Genet. Psychol. 168, 147–176. doi: 10.3200/GNTP.168.2.147-176, 17936970

[ref8] Collaborative for Academic, Social, and Emotional Learning (2017) The 2013 CASEL guide: effective social and emotional learning programs - preschool and elementary school edition Available online at: http://www.casel.org/preschool-and-elementary-edition-casel-guide/ (Accessed September 26, 2012).

[ref9] Cox M. J. Paley B. (2003). Understanding families as systems. Curr. Dir. Psychol. Sci. 12, 193–196. doi: 10.1111/1467-8721.01259

[ref10] Deng L. Tang Y. Zou S. Li B. (2024). Re-examining the issue of “good”parenting: the intensive parenting and its impact on the child development. J. Beijing Norm. Univ. Soc. Sci. 4, 57–68.

[ref11] Du Y. Wu R. Zhang B. Zhang X. Ding J. (2021). The relationship among parenting differences, sense of coherence and adolescents` psychological resilience. Chin. J. Clin. Psychol. 29, 292–296. doi: 10.16128/j.cnki.1005-3611.2021.02.015

[ref12] Egami S. (2020). “Constructing and validating a Japanese version of the intensive parenting attitude questionnaire (J-IPAQ) and investigating its relationship with parenting behavior,” in A Closer Look at Parenting Styles and Practices, ed. Roman N. V. (New York: Nova Publishers), 301–325.

[ref13] Egami S. (2024). Impact of "intensive parenting attitude" on children's social competence via maternal parenting behavior. Front. Psychol. 15:1337531. doi: 10.3389/fpsyg.2024.1337531, 38765832 PMC11100988

[ref14] Epstein N. B. Baldwin L. M. Bishop D. S. (1983). The mcmaster family assessment device. J. Marital. Fam. Ther. 9, 171–180. doi: 10.1111/j.1752-0606.1985.tb00028.x

[ref15] Fernández-Castillo A. (2020). Early childhood social competence scale (EC-SCS): factor structure and psychometric properties. Sustainability 12:6262. doi: 10.3390/su12156262

[ref16] Gauthier A. H. Bryson C. Fadel L. Haux T. Koops J. Mynarska M. (2021). Exploring the concept of intensive parenting in a three-country study. Demogr. Res. 44, 333–348. doi: 10.4054/DemRes.2021.44.13

[ref17] Guo Z. (2023). Relationship between migrant children’s parenting styles and self-control: the chain mediating role of family function and self-esteem. Stud. Psychol. Behav. 21, 503–509. doi: 10.12139/j.1672-0628.2023.04.010

[ref18] Guo Y. Liu K. Zhai Y. (2016). Correlation between parenting style and resilience in children and adolescents from divorced families. Chin. J. Sch. Health 37, 701–703, 707. doi: 10.16835/j.cnki.1000-9817.2016.05.019

[ref20] Harris K. Harris C. Dunkley L. (2022). A longitudinal investigation of the effects of parental discipline strategies on social competence in early childhood. J. Child. Educ. Soc. 3, 168–176. doi: 10.37291/2717638X.202232155

[ref21] Hays S. (1986). The Cultural Contradictions of Motherhood. New Haven: Yale University Press.

[ref22] Hou Y. Zhao J. Yang X. Zhang X. (2018). Family APGAR and self-esteem as mediators between parenting styles and life satisfaction among medical undergraduates. Chin. J. Sch. Health 39, 71–75. doi: 10.16835/j.cnki.1000-9817.2018.01.021

[ref23] Hu L. Bentler P. M. (1999). Cutoff criteria for fit indices in covariance structure analysis: conventional criteria versus new alternatives. Struct. Equ. Model. 6, 1–55. doi: 10.1080/10705519909540118.

[ref24] Huang Z. (2024). Social and Emotional Competence: Theory, Policy, and Practice. 2nd Edn Shanghai, China: East China Normal University Press.

[ref25] Huang S. Chen Q. Jia Y. Zeng L. (2021). Effect of parenting style on academic engagement of junior high school students: the mediating role of family function. J. Anshun Univ. 23, 82–86.

[ref26] Huang C. Guo L. Guo L. Guo Y. (2024). Associations of family functioning and parental styles with anxiety symptoms among high-grade primary school students. Chin. J. Sch. Health 45, 394–397, 401. doi: 10.16835/j.cnki.1000-9817.2024079

[ref27] Huang H. Wang X. (2024). The effect of mindful parenting on preschool children’s problem behaviors: the chain mediating role of child-parent relationship and psychological resilience. Psychol. Dev. Educ. 40, 533–541. doi: 10.16187/j.cnki.issn1001-4918.2024.04.08

[ref28] Ishizuka P. (2019). Social class, gender, and contemporary parenting standards in the United States: evidence from a national survey experiment. Soc. Forces 98, 31–58. doi: 10.1093/sf/soy107

[ref29] Ji Y. Niu Y. Tang Z. Yang H. (2015). Validity and reliability of the Chinese version of the Devereux early childhood assessment for preschoolers second edition. Chin. Ment. Health J. 29, 551–555.

[ref30] Jin X. Liu H. (2018). Rural migrant parenting styles and rural migrant children’s resilience: characteristics and relationships. J. Xi’an Jiaotong Univ 38, 50–59. doi: 10.15896/j.xjtuskxb.201802006

[ref31] Jones D. E. Greenberg M. Crowley M. (2015). Early social-emotional functioning and public health: the relationship between kindergarten social competence and future wellness. Am. J. Public Health 105, e1–e8. doi: 10.2105/AJPH.2015.302630, 26180975 PMC4605168

[ref32] Khademi A. Wells C. S. Oliveri M. E. Villalonga-Olives E. (2023). Examining appropriacy of cfi and tli cutoff value in multiple-group cfa test of measurement invariance to enhance accuracy of test score interpretation. SAGE Open 13. doi: 10.1177/21582440231205354

[ref33] Kılıçoğlu A. N. Güldal Ş. Kasapoğlu F. (2024). The mediating effect of mindfulness on the relationship between parental attitudes and psychological resilience in university students. HAYEF: J. Educ. 21, 2–8. doi: 10.5152/hayef.2024.22064

[ref34] Krisztián J. Barrett K. C. (2018). Affective and social mastery motivation in preschool as predictors of early school success: a longitudinal study. Early Child Res. Q. 45, 81–92. doi: 10.1016/j.ecresq.2018.05.007

[ref35] Li X. (2021). Family Function and Pathological Internet use of Middle School Students: The Mediating Role of Hope and the Regulating Role of Social Withdrawal. vol. 18. Guangzhou: Guangzhou University.

[ref36] Li M. Huang J. (2025). Intensive parenting in Western sociological studies: contexts, social consequences, and policy implications. J. Fujian Normal Univ. 6, 104–172. doi: 10.12046/j.issn.1000-5285.2025.06.010

[ref37] Li X. Lam C. B. Chung K. K. H. Cheung R. Y. M. Leung C. Fung W. K. (2020). Development and validation of the Chinese inventory of children’s socioemotional competence (CICSEC). Early Educ. Dev. 31, 854–872. doi: 10.1080/10409289.2020.1715735

[ref38] Li S. Tang Y. L. Zheng Y. (2023). How the home learning environment contributes to children's social–emotional competence: a moderated mediation model. Front. Psychol. 14:1065978. doi: 10.3389/fpsyg.2023.1234567 36865364 PMC9971822

[ref39] Li Y. Wang C. Ren L. (2024). Intensiveness of mothering in contemporary China and its relationship with preschoolers’ social-emotional development:the suppression effect of maternal psychological well-being. Studies Early Childhood Educ. 1, 61–75. doi: 10.13861/j.cnki.sece.2024.01.009

[ref40] Li S. Wen J. (2021). "intensive parenting": the transformation practice of contemporary parenting and its reflections. J. Natl. Acad. Educ. Adm. 3, 48–57.

[ref41] Li H. Zhang W. (2006). Review of the studies on psychological resilience. J. Shandong Normal Univ (Humanities and Social Sciences) 51, 149–152. doi: 10.16456/j.cnki.1001-5973.2006.03.029

[ref42] Li J. Zhang M. (2020). Income inequality, competitions in education, and the choice of family investment in education. Educ. Res. 41, 75–84.

[ref43] Liss M. Schiffrin H. H. Mackintosh V. H. Miles-McLean H. Erchull M. J. (2013). Development and validation of a quantitative measure of intensive parenting attitudes. J. Child Fam. Stud. 22, 621–636. doi: 10.1007/s10826-012-9616-y

[ref44] Liu X. Li Y. Xie Q. Xu J. Zhu J. (2020). Family functioning and preschoolers’ social adaptation: the mediating role of children's social anxiety. Chin. J. Clin. Psych. 28, 619–623. doi: 10.16128/j.cnki.1005-3611.2020.03.038

[ref45] Liu Q. Wang J. Hou L. (2023). Family cumulative risk and children's resilience: the moderation role of teacher-child relationship. Chin. J. Health Psychol. 31, 1287–1293. doi: 10.13342/j.cnki.cjhp.2023.09.002

[ref001] Long H. Prikhidko A. (2024). The mediating effect of emotion regulation between intensive parenting attitudes and parental anger. The Family Journal. 32. doi: 10.1177/10664807241257483, 22503075

[ref002] Lv C. F. Zhou Y. H. (2023). The effect of family function on problem behavior in the disadvantaged children: Moderated mediation model. J. Clin. Psychol. 31. doi: 10.16128/j.cnki.1005-3611.2023.04.041, 22503075

[ref47] Mefharet V. C. (2023). The moderating effect of parenting stress on temperament and social competence in early childhood. Curr. Psychol. 42:42. doi: 10.1007/s12144-022-03802-8

[ref48] Mesman E. Vreeker A. Hilligegers M. (2021). Resilience and mental health in children and adolescents: an update of the recent literature and future directions. Curr. Opin. Psychiatry 34, 586–592. doi: 10.1097/YCO.0000000000000741, 34433193 PMC8500371

[ref49] Miller I. W. Ryan C. E. Keitner G. I. Bishop D. S. Epstein N. B. (2010). The McMaster approach to families: theory, assessment, treatment, research. J. Fam. Ther. 22, 168–189. doi: 10.1111/1467-6427.00145

[ref51] Nokali N. E. Bachman H. J. Votruba-Drzal E. (2010). Parent involvement and children’s academic and social development in elementary school. Child Dev. 81, 988–1005. doi: 10.1111/j.1467-8624.2010.01447.x, 20573118 PMC2973328

[ref52] Olson D. H. (2000). Circumplex model of marital and family systems. J. Fam. Ther. 22, 144–167. doi: 10.1111/1467-6427.00144 6840263

[ref53] Padilla-Walker L. M. Nelson L. J. (2012). Black Hawk down? Establishing helicopter parenting as a distinct construct from other forms of parental control during emerging adulthood. J. Adolesc. 35, 1177–1190. doi: 10.1016/j.adolescence.2012.03.007, 22503075

[ref54] Peng K. Sun P. Ni S. (2022). Handbook of Positive Psychological Assessment in China. Beijing: Tsinghua University Press.

[ref55] Podsakoff P. M. MacKenzie S. B. Lee J. Y. Podsakoff N. P. (2003). Common method biases in behavioral research: a critical review of the literature and recommended remedies. J. Appl. Psychol. 88, 879–903. doi: 10.1037/0021-9010.88.5.879, 14516251

[ref56] Ryan K. M. Zimmer-Gembeck M. J. Hawes T. Kovacs T. Leahy N. (2026). Intrusive parenting and adolescent internalizing and externalizing symptoms: three-level meta-analytic reviews considering parenting concepts and methodology. Clin. Child. Fam. Psychol. Rev. 29, 105–127. doi: 10.1007/s10567-025-00555-1, 41511635 PMC12979328

[ref57] Schiffrin H. H. Godfrey H. Liss M. Erchull M. J. (2015). Intensive parenting: does it have the desired impact on child outcomes? J. Child Fam. Stud. 24, 2322–2331. doi: 10.1007/s10826-014-0035-0

[ref58] Schiffrin H. Liss M. Miles-Mclean H. Geary K. Erchull M. Tashner T. (2014). Helping or hovering? The effects of helicopter parenting on college students’ well-being. J. Child Fam. Stud. 23, 548–558. doi: 10.1007/s10826-013-9716-3

[ref59] Sell M. Daubmann A. Zapf H. Adema B. Busmann M. Stiawa M. . (2021). Family functioning in families affected by parental mental illness: parent, child, and clinician ratings. Multidisciplinary Digital Publishing Institute 18:7985. doi: 10.3390/IJERPH18157985 PMC8345719 34360277

[ref60] Shen Y. Luo H. (2023). The mechanism of parenting practices impeding the fertility intention for another child: evidence from couples with children in Shanghai. J. Univ Chinese Academy Soc. Sci. 43, 41–56+153.

[ref61] Sun X. Xu T. (2019). Relationship between family function and parenting style of mobile adolescents. Chin. J. Health Psychol. 27, 1555–1558. doi: 10.13342/j.cnki.cjhp.2019.10.028

[ref62] Sun M. Xu D. (2023). The influence of family structure on mental resilience in children with mild intellectual disabilities: the mediating role of parenting styles. Modern Special Education 22, 22–29.

[ref63] Tian G. Liu T. (2024). Research on the resilience status and improvement measures of trainee teachers from the perspective of conservation of resources theory. Teacher Development Res. 8, 88–96. doi: 10.19618/j.cnki.issn2096-319x.2024.04.013

[ref64] Townshend K. (2016). Conceptualizing the key processes of mindful parenting and its application to youth mental health. Australas. Psychiatry 24, 575–577. doi: 10.1177/1039856216654392, 27354336

[ref66] Waldman M. R. Hepworth K. Johnson J. Tourek K. M. Jones K. J. Garcia Y. E. . (2025). Validation of the Kidsights measurement tool: a parent-reported instrument to track children's development at the population level. PLoS One 20:e0324082. doi: 10.1371/journal.pone.0324082, 40569984 PMC12200676

[ref67] Wang Y. H. (2023). Deep intervention education: realistic manifestations and social roots. People's Tribune 10, 58–61.

[ref68] Wang Y. Boele S. Keijsers L. Hawk S. T. (2025). Controlling or caring? Associations between helicopter parenting and perceived conflict and support at the within-family level. J. Adolesc. 97, 1667–1679. doi: 10.1002/jad.12527, 40457959 PMC12318470

[ref69] Wang Y. Hawk S. T. (2023). Adolescent-mother agreements and discrepancies in reports of helicopter parenting: associations with perceived conflict and support. J. Youth Adolesc. 52, 2480–2493. doi: 10.1007/s10964-023-01831-5, 37542008

[ref70] Wang Q. Huang Q. Liu X. Chi X. (2020). The association between family functioning and externalizing behavior in early adolescents: the mediating effect of resilience and moderating effect of gender. Stud. Psychol. Behav. 18, 659–665.

[ref71] Wang Y. Li Y. Wu F. (2021). Effects of family function on pre-schoolers’ problem behaviors: the sequential mediating effects of attachment avoidance and social anxiety. Psychol. Dev. Educ. 37, 76–83. doi: 10.16187/j.cnki.issn1001-4918.2021.01.10

[ref72] Wang E. Xia M. Qu D. Zhang J. Liang K. Xiao J. . (2024). The effect of prototypical family functioning trajectories on junior high school students` internet addiction: the mediating role of resilience. Psychol. Dev. Educ. 40, 853–864. doi: 10.16187/j.cnki.issn1001-4918.2024.06.10

[ref73] Wen Z. Hau K. T. Marsh H. W. (2004). Structural equation model testing: cutoff criteria for goodness of fit indices and chi-square test. Acta Psychol. Sin. 36, 186–194.

[ref74] Wen S. Yu X. Jin L. Gong J. Zhang X. Sun J. . (2024). A three-level meta-analysis of the relationship between family dysfunction and mental health in children and adolescents. Adv. Psychol. Sci. 32, 771–789. doi: 10.3724/SP.J.1042.2024.00771

[ref75] Wu S. S. Pan H. Sheldrick R. C. Shao J. Liu X. M. Zheng S. S. . (2025). Development and validation of the parent-reported Indicator of developmental evaluation for Chinese children (PRIDE) tool. World J. Pediatr. 21, 183–191. doi: 10.1007/s12519-025-00878-7, 39988643

[ref76] Xie F. Gao G. Xie L. Cao H. Weng T. Wang S. . (2024). The associations between family rearing environment, family functioning, socioeconomic status, and autistic behaviors in 3-year-old children. Mod. Prev. Med. 51, 3535–3539+3550. doi: 10.20043/j.cnki.MPM.202404261

[ref77] Xing Y. Xu Y. Wang X. Zhang H. Yu X. Zhao H. (2016). The effect of family environment on preschool children’s emotional and behavioral problems: a chain mediation model through optimism and resilience. Psychol. Explor. 36, 158–163.

[ref78] Yang K. (2018). Motherhood as educational agent: changes in motherhood in the context of market-oriented education. J. Chin. Womens Stud. 2, 79–90.

[ref79] Ye P. Zhang L. Wang Q. (2023). The effects of family socioeconomic status on children’s emotional behavior: based on the analysis of chain mediation model. Educ. Sci. 2, 78–85.

[ref80] Yerkes M. A. Hopman M. Stok F. M. De Wit J. (2021). In the best interests of children? The paradox of intensive parenting and children’s health. Crit. Public Health 31, 349–360. doi: 10.1080/09581596.2019.1690632

[ref81] Yi H. (2022). Practical Drawbacks and Improvement Strategies of Intensive Family Education. Wuhan: Central China Normal University.

[ref82] Zeng S. Li Q. (2003). A review of research on the development of psychological resilience in children. J. Psychol. Sci. 26, 1091–1094.

[ref83] Zhang J. (2018). Correlation Analysis of Caregivers' Parenting Knowledge and Infants' Social-Emotional Development in Rural Areas. Xi‘an: Shaanxi Normal University.

[ref84] Zhang S. Fang F. Bai F. (2018). A study on the relationship between family cohesion and adaptability and parenting styles among college students. J. Lanzhou Institute Educ. 6, 149–150+154.

[ref85] Zhang Q. Leng L. Chen H. Fang X. Shu Z. Lin X. (2017). Parental rearing pattern mediates the association between socioeconomic status and cognitive ability of migrant children. Psychol. Dev. Educ. 33, 153–162. doi: 10.16187/j.cnki.issn1001-4918.2017.02.04

[ref86] Zhu X. Fan C. Liu Q. Zhang D. Zhou Z. (2018). Effect of bully victimization on children’s well-being: the role of resilience. Chin. J. Clin. Psychol. 2, 396–400. doi: 10.16128/j.cnki.1005-3611.2018.02.040

